# Validation of the Malay version of Epworth sleepiness scale for children and adolescents (MESS-CHAD)

**DOI:** 10.1186/s12903-023-03762-w

**Published:** 2023-12-19

**Authors:** Wan Ying Lee, May Nak Lau, Eunice Xinwei Soh, Sze Wan Yuen, Asma Ashari, Zamri Radzi

**Affiliations:** 1 Lanang Dental Clinic, Ministry of Health Malaysia, Sibu, Sarawak Malaysia; 2 Department of Paediatric Dentistry & Orthodontics, Faculty of Dentistry, Universiti Malaya, Kuala Lumpur, Malaysia; 3 Centre for Family Oral Health, Faculty of Dentistry, The National University of Malaysia, Kuala Lumpur, Malaysia

**Keywords:** Malay, ESS-CHAD, Reliability, Translation, Validity

## Abstract

**Background:**

Epworth Sleepiness Scale for Children and Adolescents (ESS-CHAD) is a valid and reliable eight-item self-administered questionnaire for the assessment of excessive daytime sleepiness and is commonly used to screen sleep-disordered breathing for children and adolescents. The cross-sectional study aimed to translate and cross-culturally adapt ESS-CHAD into a Malay version of Epworth Sleepiness Scale for Children and Adolescents (MESS-CHAD) for the Malaysian population, and to assess the validity and reliability of MESS-CHAD.

**Methods:**

Forward-backward translation method was used to translate and cross-culturally adapt ESS-CHAD. Three linguistic experts and two paediatricians content validated the translated version. Face validity was conducted through audio-recorded semi-structured in-depth interviews with 14 native Malay-speaking children and adolescents followed by thematic analysis. The revised questionnaire was then proofread by a linguistic expert. A total of 40 subjects answered the MESS-CHAD twice, 2 weeks apart, for test-retest reliability and internal consistency. For criterion validity, 148 eligible subjects and their parents answered MESS-CHAD and the Malay version of Sleep-Related Breathing Disorder scale extracted from the Paediatric Sleep Questionnaire (M-PSQ:SRBD) concurrently. Variance Inflation Factor (VIF) and *P* values of the model’s outer weight and outer loading were analysed using SmartPLS software to assess the indicator’s multicollinearity and significance for formative construct validity.

**Results:**

Intraclass Correlation Coefficient (ICC) ranging from 0.798 to 0.932 and Cronbach’s alpha ranging from 0.813 to 0.932 confirmed good to excellent test-retest reliability and internal consistency, respectively. Spearman Correlation Coefficient value of 0.789 suggested a very strong positive correlation between MESS-CHAD and M-PSQ:SRBD. VIF ranging from 1.109 to 1.455 indicated no collinearity problem. All questionnaire items in MESS-CHAD were retained as the *P* value of either outer model weight or outer model loading was significant (*P* < 0.05).

**Conclusion:**

ESS-CHAD has been translated and cross-culturally adapted into Malay version for the Malaysian population, and found to be valid and reliable.

**Supplementary Information:**

The online version contains supplementary material available at 10.1186/s12903-023-03762-w.

## Background

Excessive daytime sleepiness is caused by a wide range of sleep disorders including obstructive sleep apnoea (OSA) and various other aetiologies, with prevalence ranging from 10 to 20% in prepubertal children and 16 to 47% in adolescents [1, 2]. Paediatric obstructive sleep apnoea (OSA) has been estimated to affect about 1 to 5% of children and adolescents [1].

Untreated paediatric sleep disorders and associated excessive daytime sleepiness can result in hyperactivity, difficulties in paying attention, declining academic performance, social problems, increased risk for alcohol and drug use in teenagers, anxiety, depression, impairments in neurocognitive function, and increased risk of accidents on the streets as a pedestrian, at school, and at home [3–6]. Children and adolescents with shorter sleep duration have been correlated with an increased risk of obesity [7]. Frequent arousal during sleep may interrupt growth hormone secretion which is essential for normal growth. Multiple studies suggested that children and adolescents with sleep-disordered breathing primarily due to adenotonsillar hypertrophy are associated with an increased risk of growth failure and insulin resistance [8, 9]. More devastating morbidities, such as growth failure, pulmonary hypertension, and cor pulmonale may occur if paediatric severe obstructive sleep apnoea (OSA) is left untreated [10–12] Recent literature also suggested that sleeping disorders among children and adolescents have been significantly associated with sleep bruxism, temporomandibular disorders, and dental caries [13, 14].

Accurate diagnosis of excessive daytime sleepiness and obstructive sleep apnoea (OSA) requires a comprehensive history taking, examination, usage of screening questionnaires, and specialised sleep tests. Polysomnography (PSG), Maintenance of Wakeful Test (MWT), and Multiple Sleep Latency Test (MSLT) are performed in sleep disorder centres and used as the gold standard to diagnose excessive daytime sleepiness and obstructive sleep apnoea (OSA) [15]. However, these tests are expensive, technical-sensitive, and not widely available. Therefore, a rapid and inexpensive way to screen excessive daytime sleepiness and obstructive sleep apnoea (OSA) for children and adolescents by using validated screening questionnaires is useful to determine whether comprehensive sleep tests are indicated [16].

Murray W. Johns developed the Epworth Sleepiness Scale for Children and Adolescents (ESS-CHAD), which has been shown to be valid, reliable, and unidimensional [17]. Epworth Sleepiness Scale for Children and Adolescents (ESS-CHAD) scores exceeding 10 suggest excessive daytime sleepiness and may indicate the presence of underlying sleep disorders [18]. Furthermore, Epworth Sleepiness Scale for Children and Adolescents (ESS-CHAD) could aid in delineating the clinical and polysomnography (PSG) characteristics of sleep disorders in children with excessive daytime sleepiness [19], as well as monitoring the treatment progress of sleep disorders by measuring excessive daytime sleepiness pre- and post-treatment, e.g., for nasal continuous positive airway pressure or mandibular advancement appliance therapy [20]. To date, Epworth Sleepiness Scale for Children and Adolescents (ESS-CHAD) has been translated and validated into Persian [21], Turkish [22], and Brazilian Portugese [23].

Due to the widespread use of Epworth Sleepiness Scale for Children and Adolescents (ESS-CHAD), translation and validation in the local language are required, especially as the questionnaire is self-administered in nature. Currently, there has been no validated translation of Epworth Sleepiness Scale for Children and Adolescents (ESS-CHAD) into Malay. A validated and standardised translated version is essential as it allows for meaningful comparisons between the results of different studies and sleep clinics. Considering the importance of having a Malay version of Epworth Sleepiness Scale for Children and Adolescents (MESS-CHAD) for children and adolescents in Malaysia, this study aims to translate and cross-culturally adapt Epworth Sleepiness Scale for Children and Adolescents (ESS-CHAD) into a Malay version of Epworth Sleepiness Scale for Children and Adolescents (MESS-CHAD), and to assess the validity (content, face, criterion, and construct validity) and reliability (test-retest reliability and internal consistency) of Malay version of Epworth Sleepiness Scale for Children and Adolescents (MESS-CHAD).

## Methods

### Ethical considerations

The study protocol was approved by the Medical Ethics Committee, Faculty of Dentistry, Universiti Malaya (DF CD2019/0104 (L)). Permission to translate and use the Epworth Sleepiness Scale for Children and Adolescents (ESS-CHAD) questionnaire was obtained from the copyright holder through the Mapi Research Trust (https://eprovide.mapi-trust.org). Translation and face validity were conducted based on the Linguistic Validation Guidance of a Clinical Outcome Assessment by Mapi Research Trust.

### Instruments

#### Epworth sleepiness scale for children and adolescents (ESS-CHAD)

The study instrument used in this study was Epworth Sleepiness Scale for Children and Adolescents (ESS-CHAD) (Mapi Research Trust: https://eprovide.mapi-trust.org), a tool to measure the level of daytime sleepiness. The Epworth Sleepiness Scale for Children and Adolescents (ESS-CHAD) questionnaire consists of eight items in which respondents must rate the likelihood of dozing off in each situation on a 4-point Likert scale from 0 (would never fall asleep) to 3 (high chance of falling asleep), resulting in a final score ranging from 0 to 24 [16].

#### Malay version of sleep-related breathing disorder scale extracted from the Paediatric sleep questionnaire (M-PSQ:SRBD)

Sleep-Related Breathing Disorder scale extracted from the Paediatric Sleep Questionnaire (PSQ:SRBD) consists of 22 closed-response questionnaire items, extracted from the Paediatric Sleep Questionnaire (PSQ), and validated against polysomnography (PSG), Multiple Sleep Latency Test (MSLT) results (for the sleepiness subscale), and sleep-related breathing disorder treatment (adenotonsillectomy) outcomes. The 22 items of the Sleep-Related Breathing Disorder Scale extracted from the Paediatric Sleep Questionnaire (PSQ:SRBD) are each answered ‘yes’ = 1, ‘no’ = 0, or ‘don’t know’ = missing [24]. It has been cross-culturally adapted into the Malay version of Sleep-Related Breathing Disorder Scale extracted from the Paediatric Sleep Questionnaire (M-PSQ:SRBD) and has acceptable psychometric measurement properties as a screening tool to assess sleep-disordered breathing in Malay speaking population [25].

### Study population

This cross-sectional study was conducted with a combination of qualitative and quantitative approaches. The sample size for each phase was suggested according to references as stated in Table 1 below:

**Table 1:** Sample size for each validity test

Phases	Sample Size [Reference]
Content Validity	3 linguistic experts and 2 paediatricians [26]
Face Validity	14 children and adolescents [27]
Reliability	40 children and adolescents [28]
Criterion Validity	148 children and adolescents with their parent [29]
Construct Validity	148 children and adolescents with their parent [29]

Children and adolescents were recruited based on the following eligibility criteria for reliability, construct validity, and criterion validity tests.

- Inclusion criteria
  - Aged 10 to 17 years
  - Able to read and understand the Malay language
  - Average sleep time of at least 8 hours per night
- Exclusion criteria
  - Illiterate in Malay
  - Central active drug consumption
  - Psychiatric or neurological disabilities or diseases

### Expert panel

An expert panel consisting of a local coordinator (LWY), two orthodontists (AA, and ESX), one linguistic expert (YSW), and one expert in questionnaire translation and validation (LMN) critically reviewed the forward translations, backward translation, content validity, and face validity. Solutions were discussed until a consensus was reached.

### Forward translation

Forward translations from English to Malay were done independently by two orthodontists (ZR and AA) who were both native Malay speakers and fluent in English. Emphasis was given to translating the questionnaire conceptually rather than literally, which was culturally equivalent and appropriate for the target population. The expert panel critically reviewed the forward translations in order to create the first preliminary Malay version of Epworth Sleepiness Scale for Children and Adolescents (ESS-CHAD) [27].

### Backward translation

The first preliminary Malay version of Epworth Sleepiness Scale for Children and Adolescents (ESS-CHAD) was then backward translated into English by one backward translator who was a native English speaker, fluent in Malay, and blinded to the original Epworth Sleepiness Scale for Children and Adolescents (ESS-CHAD). The expert panel critically reviewed the first preliminary Malay version of Epworth Sleepiness Scale for Children and Adolescents (ESS-CHAD) and the backward translation in order to create the second preliminary Malay version of Epworth Sleepiness Scale for Children and Adolescents (ESS-CHAD )[27].

### Content validity

For content validity, the second preliminary Malay version of Epworth Sleepiness Scale for Children and Adolescents (ESS-CHAD) was critically reviewed by two paediatricians who manage paediatric obstructive sleep apnoea (OSA) patients and are bilingual. Besides, it is also critically reviewed by three linguistic experts who were proficient in Malay and English. These content experts critically reviewed all the questionnaire items for readability, clarity, and comprehensiveness, qualitatively, using a content validity survey form specifically designed for this purpose (Appendix A). The expert panel critically reviewed the second preliminary Malay version of Epworth Sleepiness Scale for Children and Adolescents (ESS-CHAD) according to the feedback collected from all content experts and produced the third preliminary Malay version of Epworth Sleepiness Scale for Children and Adolescents (ESS-CHAD) for the subsequent face validity test.

### Face validity with children and adolescents

**Fig. 1:**
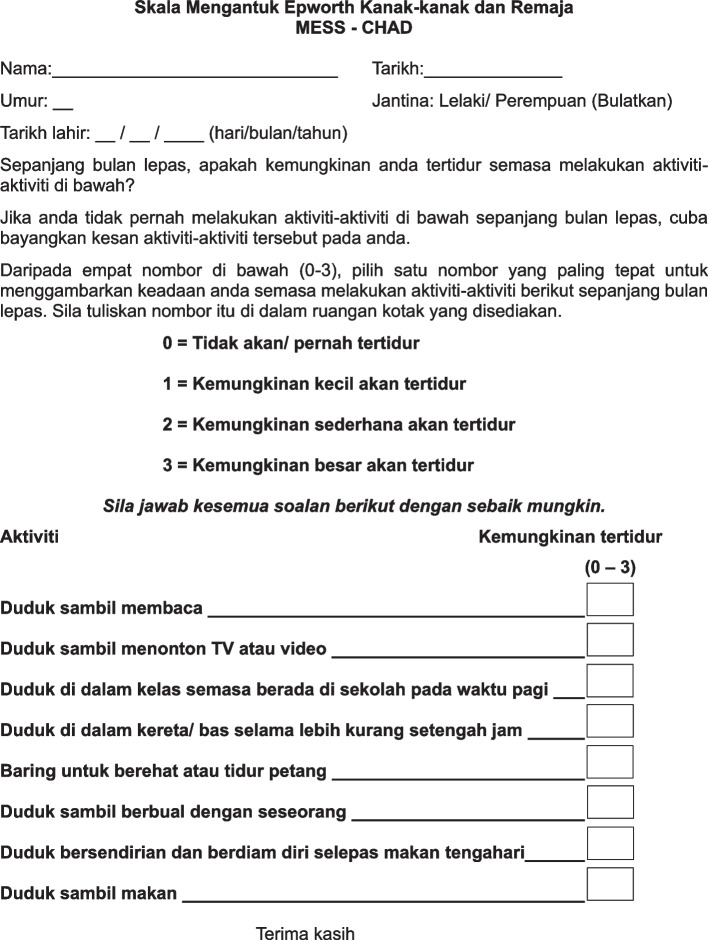
The Malay version of Epworth Sleepiness Scale for Children and Adolescents (MESS-CHAD)

Subjects who fulfilled the eligibility criteria with parents’ or legal guardians’ written informed consent for study participation were recruited for the face validity test. The third preliminary Malay version of Epworth Sleepiness Scale for Children and Adolescents (ESS-CHAD) was self-administered by 14 children and adolescents who were native Malay speakers. A single operator (LWY) conducted an audio-recorded one-on-one semi-structured in-depth interview with each respondent. The interviews were conducted in Malay, with the use of an interview guide (Appendix B), to inquire whether the participants had any difficulty in understanding and interpreting any of the questionnaire items. For any issue that arose during the interview, the interviewer either proposed or tested alternative translations (if the issue was anticipated) or requested that the participant propose alternatives. Sample recruitment for face validity was conducted until data saturation. Data were transcribed and thematically analysed, with a focus on the difficulties encountered and suggestions proposed by the respondents. The expert panel critically reviewed the third preliminary Malay version of Epworth Sleepiness Scale for Children and Adolescents (ESS-CHAD) according to the thematic analysis data and produced the fourth preliminary Malay version of Epworth Sleepiness Scale for Children and Adolescents (ESS-CHAD) for proofreading by a proofreader who was a native Malay speaker and proficient in English, specifically on any typing, spelling, or grammatical mistake to produce the final Malay version of Epworth Sleepiness Scale for Children and Adolescents (MESS-CHAD) (Fig. 1).

### Test-retest reliability and internal consistency reliability

Intraclass Correlation Coefficient (ICC) estimates and their 95% confidence intervals, were calculated using SPSS statistical package version 2 7[30]. The Intraclass Correlation Coefficient (ICC) was analysed based on an average measurement, absolute agreement, two-way mixed-effects model, which is the model of choice for test-retest/ intra-rater reliability studies [31]. The sample size calculation was derived from the Intraclass Correlation Coefficient (ICC) test formula using the same SPSS software. When alpha and power were set to 0.05 and 90%, respectively, a minimum sample size of 30 was required to detect the Intraclass Correlation Coefficient (ICC) value of 0.50. Forty eligible subjects who met the eligibility criteria with informed consent from a parent or legal guardian for study participation were recruited. Malay version of Epworth Sleepiness Scale for Children and Adolescents (MESS-CHAD) was filled out twice, 2 weeks apart. Based on the Intraclass Correlation Coefficient (ICC) guideline [31], a value of < 0.5 indicates poor reliability, 0.5 to 0.75 implies moderate reliability, 0.75 to 0.90 suggests good reliability, and > 0.90 shows excellent reliability.

Internal consistency was evaluated using Cronbach’s alpha coefficient [32], with > 0.9 indicating excellent reliability, > 0.8 indicating good reliability, > 0.7 indicating acceptable reliability, > 0.6 indicating questionable reliability, > 0.5 indicating poor reliability, and ≤ 0.5 indicating unacceptable reliability.

### Criterion validity

Malay version of Sleep-Related Breathing Disorder Scale extracted from the Paediatric Sleep Questionnaire (M-PSQ:SRBD) was used in the criterion validity to compare the ability of Malay version of Epworth Sleepiness Scale for Children and Adolescents (MESS-CHAD) with Malay version of Sleep-Related Breathing Disorder Scale extracted from the Paediatric Sleep Questionnaire (M-PSQ:SRBD) in predicting sleep-disordered breathing among children and adolescents [25]. A total of 148 eligible subjects with written informed consent from a parent or legal guardian for study participation answered both the Malay version of Epworth Sleepiness Scale for Children and Adolescents (MESS-CHAD) and Malay version of Sleep-Related Breathing Disorder Scale extracted from the Paediatric Sleep Questionnaire (M-PSQ:SRBD) concurrently. Since normality testing showed that the data was not normally distributed, the Spearman Correlation Coefficient was used to analyse the data for criterion validity with 0.01 to 0.19 suggesting no or negligible correlation, 0.20 to 0.29 showing weak correlation, 0.30 to 0.39 indicating moderate correlation, 0.40 to 0.69 implying strong correlation, and > 0.70 portraying very strong correlation [33].

### Construct validity

Construct validity was conducted based on formative measurement model using SmartPLS with Partial Least Squares Structural Equation Modeling (PLS-SEM) techniques [34] as the direction of causality was from indicators to construct and all indicators were not interchangeable and not highly correlated. A minimum sample size of 84 is required to carry out a formative measurement model with 8 arrows (questionnaire items) pointing at a latent variable [34] and a total of 148 eligible subjects were recruited.

Collinearity of each questionnaire item was analysed using Variance Inflation Factor (VIF) value. Variance Inflation Factor (VIF) value lower than 5 indicates no collinearity problem [34]. Bootstrapping was used to test significance of the formative indicators’ outer weight and outer loading. The indicator was included in the Malay version of Epworth Sleepiness Scale for Children and Adolescents (MESS-CHAD) if either outer weight or outer loading was significant (*P* < 0.05, 34].

## Results

### Descriptive statistics

Table 2 shows the distributions of participants’ basic characteristics.

**Table 2:** Subjects’ demographic characteristics (*n* = 155)

Characteristics	Study Phases		
	Face Validity,	Reliability,	Construct and Criterion Validity,
	***N*** = 14	***N*** = 40	***N*** = 148
**Age(year), Mean + SD**	13.00 + 2.42	13.70 + 2.45	14.47 + 2.13
**Gender, N (%)**			
Male	4 (29.00)	15 (37.50)	68 (45.90)
Female	10 (71.00)	25 (62.50)	80 (54.10)
**Race, N (%)**			
Malay	11 (79.00)	25 (62.50)	51 (34.50)
Indigenous group	3 (21.00)	14 (35.00)	51 (34.50)
Chinese	0 (0.00)	1 (2.50)	46 (31.10)

*N* number of participants, *SD* standard deviation.

### Forward translation, backward translation and content validity

First (Forward Translation) and second (Backward Translation), and preliminary Malay versions of Epworth Sleepiness Scale for Children and Adolescents (ESS-CHAD) were produced prior to the content validity test.

One of the linguistic experts (TSP) who works as primary school teacher suggested to change “Jantina: Lelaki () Perempuan () Pangkah (X) 1 sahaja” to “Jantina: Lelaki/ Perempuan (Bulatkan)” to allow the subjects to circle instead of putting a cross when selecting their gender which might confuse the younger children. The instructions of the questionnaire, description of scale and questionnaire item 3 were modified for clarity. For scale description, “/ pernah” was added to “0= tidak akan tertidur”. For questionnaire item 3, the word “berada” was added to “duduk di dalam kelas semasa di sekolah pada waktu pagi”. Both linguistic expert (TSP) and paediatrician (NRL) suggested to change the numerical “1” to the alphabetical “satu”. The third preliminary Malay version of Epworth Sleepiness Scale for Children and Adolescents (ESS-CHAD) after content validity was produced.

### Face validity

The cognitive interviewing procedures for face validity with children and adolescents revealed that all respondents knew that the purpose of this questionnaire was to inquire about the possibility of falling asleep while performing the activities stated. They had done all of the activities stated, albeit not exactly 1 month ago. For the activities that were not done 1 month ago, they managed to explain that they filled out the questionnaire based on previous experience. Respondents managed to explain that they chose score “0” when they would not fall asleep; score “1” when they felt a slight possibility of falling asleep; score “2” when they felt a moderate possibility of falling asleep; score “3” when they felt a high possibility of falling asleep.

Some respondents claimed that they did not understand the terms “Skala” and “Epworth” in the title. When asked to explain the word “Skala”, four of them did not understand, two understood but were unable to articulate the meaning. However, after reading the instructions, they managed to complete the questionnaire without parental assistance and explain what it was about. We opted to keep the questionnaire title “Skala Mengantuk Epworth” because it had no effect on children’s ability to answer the following questions. Instructions of MESSCHAD was simplified from “menggunakan skala yang disediakan” to “Daripada empat nombor di bawah (0-3)”.

Two children were confused with the word “kemungkinan”, yet they understood “mungkin”. We decided to retain the word “kemungkinan” because “mungkin” meant “maybe” whereas “kemungkinan” meant “possibility”. The connotation of “slight chance” would be lost in the translated sentence if “kemungkinan” was replaced with “mungkin”.

Two 10-year-old respondents misread the instructions and filled in “umur: 10 (tahun) 4” as they were in Primary 4. We removed the word “(tahun)” to minimise confusion among younger children. In addition, “tarikh lahir: __ / __ / ____ (hari/bulan/tahun)” was included to determine the exact age of children.

Otherwise, all respondents denied having any issues answering the questionnaire, answered that none of the words or expressions was found unaccepted/ offensive, and made no suggestions for improvement.

The proofreader made no changes to the fourth preliminary Malay version of Epworth Sleepiness Scale for Children and Adolescents (ESS-CHAD) (Face Validated). Table 3 shows the final Malay version of Epworth Sleepiness Scale for Children and Adolescents (MESS-CHAD) against the original Epworth Sleepiness Scale for Children and Adolescents (ESS-CHAD).

**Table 3:** The original Epworth Sleepiness Scale for Children and Adolescents (ESS-CHAD) versus final Malay version of Epworth Sleepiness Scale for Children and Adolescents (MESS-CHAD)

No	English version	Malay version
1	Sitting and reading	Duduk sambil membaca
2	Sitting and watching TV or a video	Duduk sambil menonton TV atau video
3	Sitting in a classroom at school during the morning	Duduk di dalam kelas semasa berada di sekolah pada waktu pagi
4	Sitting and riding in a car or bus for about half an hour	Duduk di dalam kereta/ bas selama lebih kurang setengah jam
5	Lying down to rest or nap in the afternoon	Baring untuk berehat atau tidur petang
6	Sitting and talking to someone	Duduk sambil berbual dengan seseorang
7	Sitting quietly by yourself after lunch	Duduk bersendirian dan berdiam diri selepas makan tengahari
8	Sitting and eating a meal	Duduk sambil makan

### Test-retest reliability and internal consistency reliability

Intraclass Correlation Coefficient (ICC) and Cronbach’s Alpha of 0.932 for the total Malay version of Epworth Sleepiness Scale for Children and Adolescents (MESS-CHAD) score suggest excellent test-retest reliability and internal consistency reliability. For each Malay version of Epworth Sleepiness Scale for Children and Adolescents (MESS-CHAD) item, the Intraclass Correlation Coefficient (ICC) ranged from 0.798 to 0.916 and the Cronbach’s alpha ranged from 0.813 to 0.917, indicating good to excellent test- retest reliability and internal consistency, respectively (Table 4).

**Table 4:** Psychometric properties of Malay version of Epworth Sleepiness Scale for Children and Adolescents (MESS-CHAD) in item level

MESS-CHAD Item	Reliability		Construct Validity		
	Intraclass Correlation Coefficient (ICC)	Cronbach’s Alpha	P values of Outer Model		Variance Inflation Factor (VIF)
			Weight	Loading	
Q1	0.798	0.828	0.000	0.000	1.323
Q2	0.818	0.816	0.016	0.000	1.328
Q3	0.822	0.818	0.003	0.000	1.226
Q4	0.853	0.850	0.000	0.000	1.109
Q5	0.911	0.909	0.004	0.000	1.165
Q6	0.817	0.813	0.100	0.000	1.455
Q7	0.831	0.842	0.002	0.000	1.257
Q8	0.916	0.917	0.009	0.000	1.391
**Total score**	**0.932**	**0.932**			

### Criterion validity

For criterion validity, Spearman Correlation Coefficient value of 0.789 suggested a very strong positive correlation between Malay version of Epworth Sleepiness Scale for Children and Adolescents (MESS-CHAD) and Malay version of Sleep-Related Breathing Disorder Scale extracted from the Paediatric Sleep Questionnaire (M-PSQ:SRBD).

### Construct validity

**Fig. 2:**
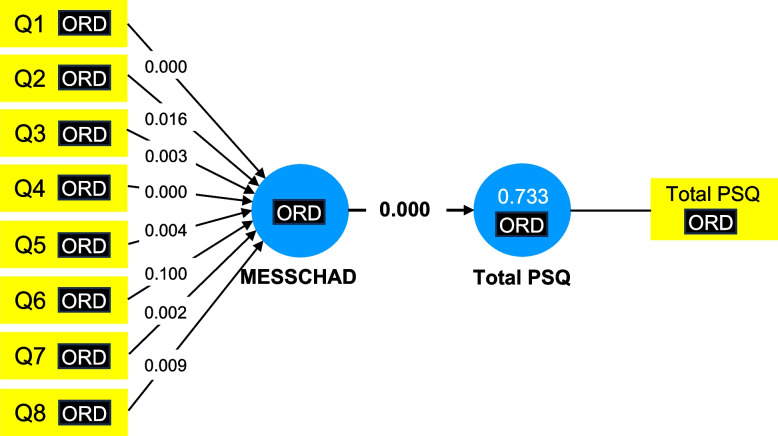
Path graph of Malay version of Epworth Sleepiness Scale for Children and Adolescents (MESS-CHAD) for construct validity of formative measurement model

Figure 2 shows the path graph that was obtained from the formative measurement model. All questionnaire items in Malay version of Epworth Sleepiness Scale for Children and Adolescents (MESS-CHAD) were retained as the *P* value of either outer model weight or outer model loading was significant (*P* < 0.05) (Table 4). Variance Inflation Factor (VIF) ranged between 1.109 and 1.455 indicating no collinearity problem (Table 4).

## Discussion

### The need for Malay version of Epworth sleepiness scale for children and adolescents (MESS-CHAD)

The prevalence of excessive daytime sleepiness has been reported to affect 40.5-58.0% of adults with obstructive sleep apnoea (OSA), depending on the severity, at initial diagnosis [35]. Even though obstructive sleep apnoea (OSA) is known to cause excessive daytime sleepiness in adults, the degree of excessive daytime sleepiness among children with obstructive sleep apnoea (OSA) remains controversial. The prevalence of excessive daytime sleepiness in children with obstructive sleep apnoea (OSA) varies widely, ranging from 7 to 43% depending on the survey methods [36, 37].

Daytime sleepiness in children and adolescents can be measured objectively or subjectively using a variety of methods. The Multiple Sleep Latency Test (MSLT) and the Maintenance of Wakeful Test (MWT) measure daytime sleepiness objectively, whereas the Epworth Sleepiness Scale for Children and Adolescents (ESS-CHAD), Paediatric Sleep Questionnaire (PSQ), Sleep Disturbance Scale for Children, and Paediatric Daytime Sleepiness Scale measure daytime sleepiness in children and adolescents subjectively.

Subjective measurement of sleepiness can serve as a useful screening tool for paediatric sleep-disordered breathing, such as paediatric obstructive sleep apnoea (OSA). The Epworth Sleepiness Scale for Children and Adolescents (ESS-CHAD) is also useful in the management of mental and physical health in adolescents [19], and it is one of the most important screening tools for paediatric narcolepsy symptoms [38].

A recent study suggested a possible genetic predisposition to the association between sleep bruxism and obstructive sleep apnoea (OSA )[39]. Polysomnographic (PSG) data show that 49.7% of obstructive sleep apnoea (OSA) patients presented with sleep bruxism [40]. A relationship between sleep bruxism and pain-related temporomandibular disorders (TMD) was supported [41].

Since there is evidence suggesting an association between temporomandibular disorders (TMD) and sleep quality, screening for excessive daytime sleepiness may play an important role in identifying patients with sleep disturbances, enabling specialised care, and ensuring more effective assistance through multimodal management among temporomandibular disorders (TMD) patient population [17, 42]. 

The Epworth Sleepiness Scale for Children and Adolescents (ESS-CHAD) has been translated into Persian and Turkish, and shown to be a valid and reliable tool for assessing daytime sleepiness [21, 22]. There are two aspects to the importance of a translated questionnaire. From the perspective of research, translation is the most common strategy for developing methods for cross-cultural research [43]. Translated versions allow cross-cultural comparisons of the collected data from different geographies with diverse concepts, cultures, and social behaviours. From the standpoint of clinical practicality, a translated and validated questionnaire enables a valid and reliable clinical information acquisition for a targeted community that is literate in the target language, especially for self-administered questionnaires like Epworth Sleepiness Scale for Children and Adolescents (MESS-CHAD). Therefore, a Malay translation of the Epworth Sleepiness Scale for Children and Adolescents (ESS-CHAD) will benefit both researchers and clinicians in acquiring data from Malay-speaking children and adolescents in Malaysia.

### Face validity approach

The face validity of Malay version of Epworth Sleepiness Scale for Children and Adolescents (MESS-CHAD) was tested using cognitive debriefing. Cognitive debriefing is a structured interviewing technique used to improve the face validity of a questionnaire [44]. Willis (1994) developed three methods to reveal the cognitive processes that occur as respondents ponder and create responses to a questionnaire. Firstly, respondents were encouraged to “think aloud” by verbalising their thoughts and explaining their reasoning behind each response. This method serves to minimise the possibility of the interviewer introducing bias into the response and to recognise how a certain topic can be problematic. Secondly, in response to the question “Can you explain to me what this statement means to you in your own words?” respondents were asked to “paraphrase” or re-state the text in their own words. The third method is probing, which involves asking a series of questions to elicit additional information from the interviewee. “Are there any words or expressions in the question that you did not understand or find confusing?” “Can you think of a better suggestion for the alternative word(s)/ expression(s) so that it would be clearer?” Cognitive debriefing was conducted in every aspect of the questionnaire, including the title, instructions, and responses.

### Reliability of Malay version of Epworth sleepiness scale for children and adolescents (MESS-CHAD)

The total scores of both Intraclass Correlation Coefficient (ICC) and Cronbach’s alpha were 0.932, respectively, implying strong test-retest reliability and internal consistency reliability in measuring excessive daytime sleepiness for children and adolescents between 10 to 17 years old, which was consistent with the results from the original Australian English, Persian, and Turkish versions which validated against adolescents between 12 to 18 years old [17, 21, 22]. Our study supports the recommendation by Epworth Sleepiness Scale for Children and Adolescents (ESS-CHAD)‘s author that children over the age of 9 years can answer the questionnaire without assistance [16].

### Criterion validity and the use of Malay version of sleep-related breathing disorder scale extracted from the Paediatric sleep questionnaire (M-PSQ:SRBD)

The positive correlation between Malay version of Epworth Sleepiness Scale for Children and Adolescents (MESS-CHAD) scores and Malay version of Sleep-Related Breathing Disorder Scale extracted from the Paediatric Sleep Questionnaire (M-PSQ:SRBD) provides initial support that the Malay version of Epworth Sleepiness Scale for Children and Adolescents (MESS-CHAD) is valid to screen for sleep-disordered breathing in children and adolescents subjectively, just like Malay version of Sleep-Related Breathing Disorder Scale extracted from the Paediatric Sleep Questionnaire (M-PSQ:SRBD). In the validation of the Iranian version of Epworth Sleepiness Scale study, there was only a poor to moderate correlation between Iranian Epworth Sleepiness Scale items and the total score of Multiple Sleep Latency Test (MSLT) results [45]. These findings suggest that future studies should look into the correlation between Malay version of Epworth Sleepiness Scale for Children and Adolescents (MESS-CHAD) which subjectively evaluates sleepiness and Multiple Sleep Latency Test (MSLT), which is an objective measure of daytime sleepiness.

### Construct validity using a formative measurement approach

The current study was the first to conduct the construct validity of Malay version of Epworth Sleepiness Scale for Children and Adolescents (MESS-CHAD) by applying a formative measurement approach, as opposed to a reflective measurement approach. This is because all questionnaire items in Malay version of Epworth Sleepiness Scale for Children and Adolescents (MESS-CHAD) and Epworth Sleepiness Scale for Children and Adolescents (ESS-CHAD) are not interchangeable and not highly correlated. Thus, conventional psychometric methods relying on the reflective model, may not be suitable for evaluating the structural validity of the questionnaire. Using exploratory factor analysis and Rasch analysis, the Epworth Sleepiness Scale for Children and Adolescents (ESS-CHAD) in English has been reported as an internally valid, reliable, and uni-dimensional questionnaire for measuring daytime sleepiness among adolescents [17]. In previous validation studies, the fit indices such as chi square/df, RMSEA, RMR, CFI, GFI, and NFI calculated as a result of confirmatory factor analysis showed model fits very well in Turkish and Persian versions [21, 22]. In our study, all questionnaire items were retained as the *P* value of either outer model weight or outer model loading was significant and the Variance Inflation Factor (VIF) indicating no collinearity problem.

Nevertheless, validation of an instrument is an ongoing process, and the applicability of the Malay version of Epworth Sleepiness Scale for Children and Adolescents (MESS-CHAD) still needs to be tested in other settings, for example, to investigate whether it is sensitive enough to detect responsiveness of Malay version of Epworth Sleepiness Scale for Children and Adolescents (MESS-CHAD) total score before and after treating obstructive sleep apnoea (OSA) such as via tonsillectomy or continuous positive airway pressure (CPAP) treatment [45].

### Limitations

The study acknowledged some limitations. Firstly, content and face validity tests were assessed qualitatively rather than quantitatively. Secondly, criterion validity was tested against the Malay version of Sleep-Related Breathing Disorder Scale extracted from the Paediatric Sleep Questionnaire (M-PSQ:SRBD) which measures daytime sleepiness subjectively instead of objectively by using Multiple Sleep Latency Test (MSLT). Thirdly, the Malay version of Epworth Sleepiness Scale for Children and Adolescents (MESS-CHAD) has not been validated using discriminant validity against the Apnoea-Hypopnoea Index (AHI) score from polysomnography (PSG) for obstructive sleep apnoea (OSA). Future studies should look into these objective evaluations of excessive daytime sleepiness and obstructive sleep apnoea (OSA), as well as their relationship with self-reported subjective evaluation using Malay version of Epworth Sleepiness Scale for Children and Adolescents (MESS-CHAD).

## Conclusion

The translated and cross-cultural adapted 8-item Malay version of Epworth Sleepiness Scale for Children and Adolescents (MESS-CHAD) showed excellent test-retest reliability and internal consistency. There was a very strong positive correlation between Malay version of Epworth Sleepiness Scale for Children and Adolescents (MESS-CHAD) and Malay version of Sleep-Related Breathing Disorder Scale extracted from the Paediatric Sleep Questionnaire (M-PSQ:SRBD) suggesting a favourable predictive validity of Malay version of Epworth Sleepiness Scale for Children and Adolescents (MESS-CHAD) to screen sleep-disordered breathing for children and adolescents subjectively. Formative measurement models supported the use of this adaptation as each questionnaire item demonstrated no collinearity problem and all questionnaire items were retained. In conclusion, Epworth Sleepiness Scale for Children and Adolescents (ESS-CHAD) has been translated and cross-culturally adapted into Malay version for the Malaysian population, and found to be valid and reliable.

### Supplementary Information

**Additional file 1: Appendix A**. Content Validity Survey Form. **Appendix B**. Face Validity Survey Form.

## Data Availability

The authors prefer not to publicly disclose the dataset because the participants in the study consented to the use of their responses by the authors for this specific study only. Sharing this information publicly would violate the consent. However, if any of the reviewers or journal editors would like to view the dataset, it is available from corresponding author on reasonable request.

## Abbreviations

ESS: Epworth Sleepiness Scale
ESS-CHAD: Epworth Sleepiness Scale for Children and Adolescents
MESS-CHAD: Malay version of Epworth Sleepiness Scale for Children and Adolescents
PSQ: Paediatric Sleep Questionnaire
SRBD: Sleep-Related Breathing Disorder Scale extracted from the Paediatric Sleep Questionnaire
M-PSQ: Malay version of Sleep-Related Breathing Disorder scale extracted from the Paediatric Sleep Questionnaire
OSA: Obstructive sleep apnoea
AHI: Apnoea-Hypopnoea Index
PSG: Polysomnography
MWT: Maintenance of Wakeful Test
MSLT: Multiple Sleep Latency Test
VIF: Variance Inflation Factor
ICC: Intraclass Correlation Coefficient
SD: Standard deviation
