# Supplementary material for: Validation of the Malay version of Epworth sleepiness scale for children and adolescents (MESS-CHAD)
Source: BMC Oral Health. 2023 Dec 19;23:1015. doi: 10.1186/s12903-023-03762-w (PMC10729393; doi:10.1186/s12903-023-03762-w)
Supplement: Supplementary file 1 — Additional file 1: Appendix A. Content Validity Survey Form. Appendix B. Face Validity Survey Form. [file 12903_2023_3762_MOESM1_ESM.docx]

**Appendix A: Content Validity Survey Form**

1. Any comment(s) on grammar of MESS-CHAD?

__________________________________________________________________________________________________________________________________________________________________________________________

**Sample Copy
Do not use without permission**

1. Any comment(s) on correct and proper order of words?

__________________________________________________________________________________________________________________________________________________________________________________________

1. Additional comment(s) and suggestion(s), e.g. on readability, clarity and comprehensiveness.

__________________________________________________________________________________________________________________________________________________________________________________________

**Appendix B: Face Validity Survey Form**

1. What do you think the questionnaire is asking?
2. Have you done all the activities listed here before? If “yes”, skip question below.
3. If “no”, how did you answer the questions?
4. Can you explain to me how you choose your answer?
5. Are there any difficulties you faced when you answer the questionnaire?
6. Are there any words or phrases that you did not understand or find confusing?

**Sample Copy
Do not use without permission**

1. If “yes”, do you have any suggestion for the alternative word(s)/ expression(s) if any?
2. Are there any words or expressions that you find unacceptable/ offensive?
3. If “yes”, do you have any suggestion for the alternative word(s)/ expression(s) if any?
4. Do you have any other suggestion(s) for improvement?
